# Conducting risk assessments and case detection in online environments in the scope of fight with COVID-19: A good practice example diabetes: A cross-sectional study

**DOI:** 10.14744/nci.2022.92979

**Published:** 2022-04-18

**Authors:** Suayip Birinci, Mustafa Mahir Ulgu, Sahin Aydin, Eray Ozcan

**Affiliations:** 1 Vice-Minister, Ministry of Health of Turkey, Ankara, Turkey; 2 Acting Director-General, SBSGM, Ankara, Turkey; 3 Deputy Director-General, SBSGM, Ankara, Turkey; 4 Software Director, SBSGM, Ankara, Turkey

**Keywords:** COVID-19, mobile health, risk assessment

## Abstract

Turkey’s Corona Precaution Application, a web-based and mobile service, has been actively used by the citizens of the Republic of Turkey and foreigners coming to Turkey since March 19, 2020. This article examines the Corona Precaution Application in terms of its success in detecting risky and positive cases among users. In this informative process analysis study, which is conducted in the lights of Ministry of Health of Turkey data, the efficiency of Corona Precaution Application in March 2020-August 2020 has been measured through the usage statistics from specific provinces and the effect of the application has been proved. The application was used by a total of 2.159.903 people on mobile and web platforms and risk assessments were made. As a result, 135.277 people who were scored as high risk were referred to health-care facilities, and 12.067 people were hospitalized with a positive diagnosis of COVID-19 in PCR tests or isolated at home. When evaluated cumulatively, Corona Precaution Application is used as an effective tool of the health system in the fight against COVID-19. 12.067 people were found to be positive with the referrals to the healthcare facility made through the application; thus, it has been one of the most effective tools in controlling the spread of the disease.

**F**ollowing the emergence of the corona virus in the People’s Republic of China, it was declared a pandemic by the World Health Organization on 11 March 2020 [1]. The atmosphere of panic that emerged on a global scale, as well as the protectionist policies of countries in information sharing, led many countries to different solution alternatives. Especially in countries where health data is used quickly and effectively, digital solutions have become the basis of the fight against COVID-19.

Since the symptoms of the disease in the first stage of the fight against COVID-19 are similar to many seasonal diseases, it has also revealed the necessity of struggling with the psychological effects of the disease before the disease itself. To eliminate the panic atmosphere in the society and to use the health service providers much more efficiently, applications similar to the triage application used especially in the emergency services of health institutions were implemented as web-based and mobile for symptom control in the fight against COVID-19.

In Turkey, the “Corona Precaution Application” was put into service by General Directorate of Health Information Systems, Ministry of Health, on March 19, 2020, 8 days after the first COVID-19 case was seen. It serves as a mobile application in IOS and Android markets, and on the web at the address koronaonlem.saglik.gov.tr (Fig. 1).

**Figure 1.**
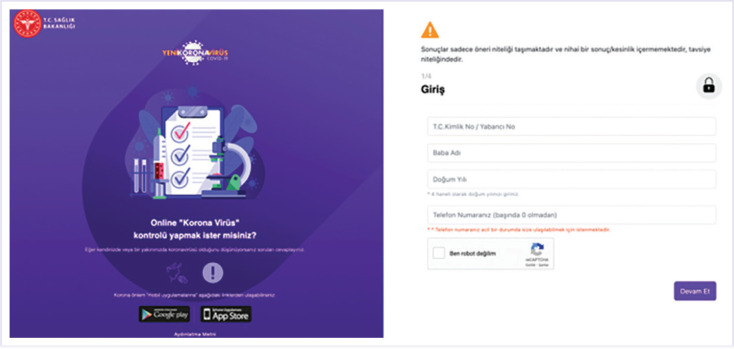
Corona Precaution Application, web home page.

The Corona Precaution Application, web-based and mobile, has been actively used by the citizens of the Republic of Turkey and foreigners coming to Turkey since 19 March 2020.

With this system, which was prepared in the light of algorithms approved by the Scientific Committee, citizens are provided to evaluate themselves on risky diseases and symptoms without the need to consult a physician by answering the questions posed to them.

With the COVID-19 pre-assessment application, which can be easily accessed and used by everyone through “koronaonlem.saglik.gov.tr” website and mobile application, the answers to the questions directed to the users are evaluated with the scoring system, and the health risk status of the person is determined and referrals to what needs to be done are made in the form of recommendations.

The first of these questions asks what age range the user is in. It is among the important information whether the person works in the health sector or not, and whether he or she contacts patients while working. In addition, other questions related to conditions that cause more severe symptoms are asked such as presence of chronic lung disease, diabetes, high blood pressure (hypertension), chronic liver disease, chronic kidney disease, chronic heart disease, genetic disorder, blood cancer (hematological cancer), other types of cancer (solid cancer), and chemotherapy (Fig. 2). Since these conditions and some others such as immune system disorder, taking cortisone treatment cause the disease to progress more severely, the system asks the user additional questions and the answers are evaluated in terms of risk detection with certain score intervals.

**Figure 2.**
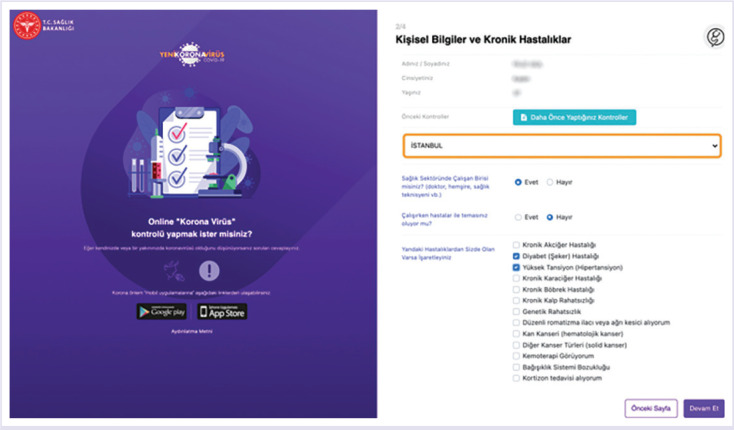
Corona Precaution Application, “Chronic Diseases” questions page.

If the person has just arrived from abroad, information is requested about the countries visited in the last 14 days, when the person entered Turkey, whether he or she was in a healthcare center in the past 14 days, and whether he or she had contact with someone with a respiratory disease (cold, flu, pneumonia, etc.) in the past 14 days (Fig. 3).

**Highlight key points**

• Ministry of Health of Turkey has been one of the first global responders to COVID-related diagnosis and symptom check problems through developing the prominent Corona Precaution application.
• Corona Precaution application has been developed within eight days, right after the WHO’s declaration of COVID-19 as a pandemic, which signifies the digitalization capacity of the Ministry of Health of Turkey.
• More than 2 million people have used the application and approximately 150.000 risky cases have been detected through the Corona Precaution application.

**Figure 3.**
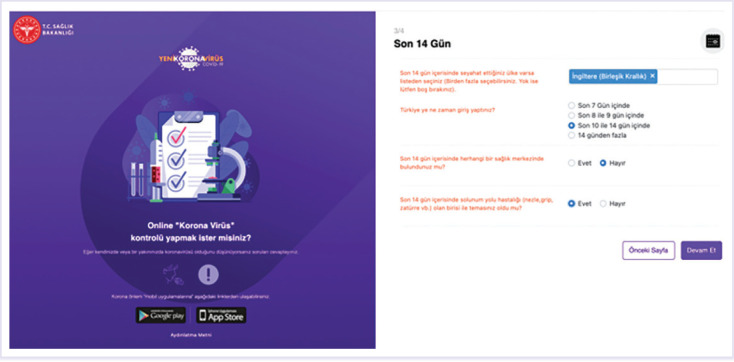
Corona Precaution Application, “last 14 days” questions page.

Current symptoms are defined by and scored with the opinion of the relevant experts in terms of evaluation as new onset cough and/or dry cough, new onset shortness of breath (breathing faster than normal, not breathing enough, and not being able to climb stairs comfortably), sore throat and headache, new onset chest tightness, new onset runny nose, new onset body aches (muscle/joint pain), new onset diarrhea, nausea or vomiting, new onset fatigue (last 1 week), and scored with the opinion of relevant (Fig. 4).

**Figure 4.**
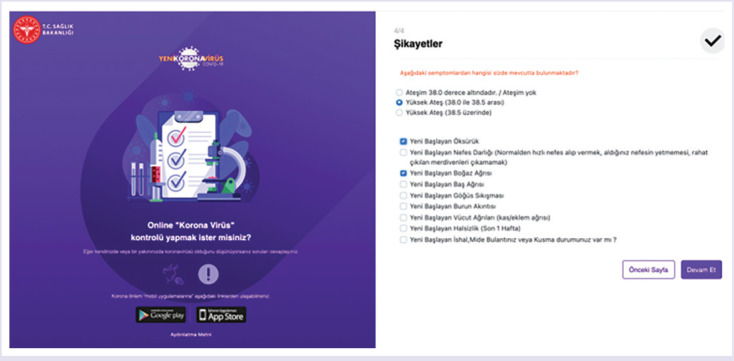
Corona Precaution Application, “symptoms” questions page.

According to the answers given, the COVID-19 risk status of the person is evaluated and the system guides the citizens according to the result. For example, if the assessment proves to be high risk the person is directed to visit a healthcare facility (Fig. 5).

**Figure 5.**
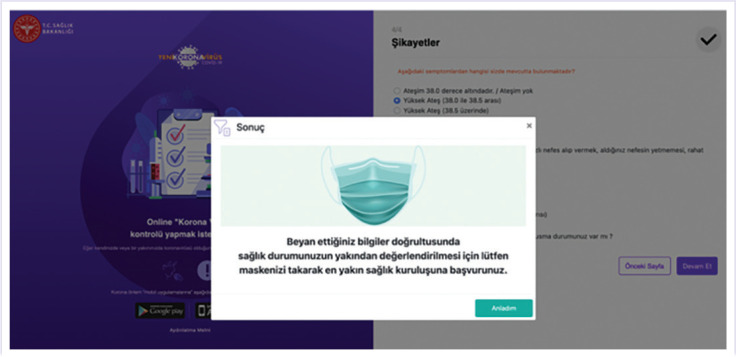
Corona Precaution Application, result page.

When we look at similar applications in the world, a similar application was put into service by the Centers for Disease Control and Prevention on March 21, 2020, in the United States [2], and another one was put into service on March 25, 2020, in Australia [3].

## The Role of the Corona Precaution Application in the Fight Against COVID-19

The Corona Precaution Application, which was put into practice by Ministry of Health on 19 March 2020, immediately after the World Health Organization announced the COVID-19 pandemic, started to be used intensively by the citizens of the Republic of Turkey. On the first day of its rollout, 309.305 people made a risk assessment on the basis of symptom control.

**Figure 6.**
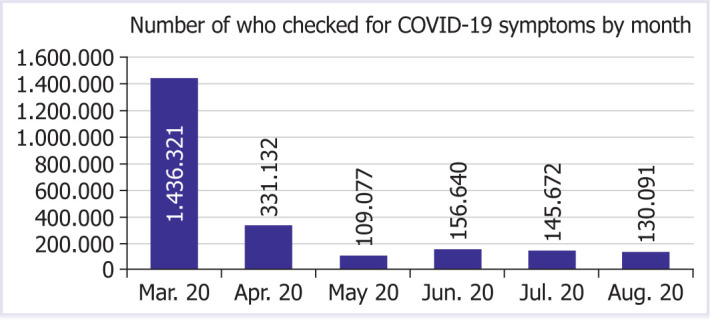
Number of people who checked for COVID-19 symptoms by month.

After the Corona Precaution Application was put into service, it was used by 1.436.321 people, especially in March 2020, when the pandemic was on the rise in the world. In the first 6 months of its rollout, a total of 2.159.903 people made risk assessments for the symptoms of Coronavirus in mobile and web environments (Fig. 6).

**Figure 7.**
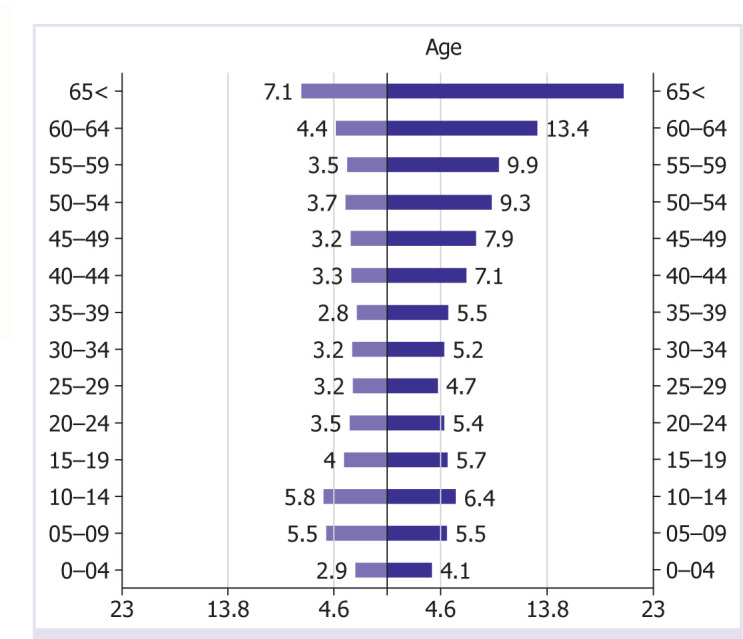
Rates of high-risk people by age and gender groups.

In the risk assessment made by users according to age groups and gender in the Corona Precaution Application, the rate of those who are considered to be high risk, especially in the population over the age of 65, draws attention (Fig. 7).

Looking at the distribution of those who filled out the COVID-19 risk assessment form using the application, Istanbul ranks first among the top ten cities. Looking at the results of the risk assessment survey, it is seen that the highest risk ratio is in Gaziantep with 5.6. On the other hand, Bursa was found to be the lowest province with a high-risk ratio of 4.0 in risk assessment surveys (Table 1).

**Table 1.** Scores of the risk assessment

City	Number of participants	Rate of high risk (%)	Rate of low/no risk (%)
Istanbul	866.002	5.0	95.0
Ankara	298.174	4.4	95.6
Izmir	170.793	4.3	95.7
Bursa	117.963	4.0	96.0
Kocaeli	86.286	4.2	95.8
Antalya	81.673	4.3	95.7
Konya	81.287	4.4	95.6
Adana	66.375	5.0	95.0
Gaziantep	64.661	5.6	94.4
Kayseri	56.767	4.8	95.2

When evaluated cumulatively, Corona Precaution Application is used as an effective tool of the health system in the fight against COVID-19. The application was used by a total of 2.159.903 people on mobile and web platforms and risk assessments were made. As a result, 135.277 people who were scored as high risk were referred to healthcare facilities, and 12.067 people were hospitalized with a positive diagnosis of COVID-19 in PCR tests or isolated at home.

The Corona Precaution Application, which was put into practice immediately after the first COVID-19 case in our country, is a platform where citizens make risk assessments for themselves and their relatives whenever they want. In addition, 12.067 people were found to be positive with the referrals to the health-care facility made through the application; thus, it has been one of the most effective tools in controlling the spread of the disease.
